# Association of Urinary Cadmium with Mortality in Patients at a Coronary Care Unit

**DOI:** 10.1371/journal.pone.0146173

**Published:** 2016-01-07

**Authors:** Ching-Wei Hsu, Cheng-Hao Weng, Dan-Tzu Lin-Tan, Pao-Hsien Chu, Tzung-Hai Yen, Kuan-Hsing Chen, Chung-Yin Lin, Wen-Hung Huang

**Affiliations:** 1 Department of Nephrology, Division of Clinical Toxicology, Chang Gung Memorial Hospital, Taipei, Taiwan; 2 Department of Nephrology, Division of Clinical Toxicology, Lin-Kou Medical Center, Taoyuan, Taiwan; 3 Chang Gung University and School of Medicine, Taoyuan, Taiwan; 4 Division of Cardiology, Chang Gung Memorial Hospital, Lin-Kou Medical Center, Taoyuan, Taiwan; National Cardiovascular Center Hospital, JAPAN

## Abstract

**Background:**

Determine the effect of the day 1 urinary excretion of cadmium (D1-UE-Cd) on mortality of patients admitted to a coronary care unit (CCU).

**Methods:**

A total of 323 patients were enrolled in this 6-month study. Urine and blood samples were taken within 24 h after CCU admission. Demographic data, clinical diagnoses, and hospital mortality were recorded. The scores of established systems for prediction of mortality in critically ill patients were calculated.

**Results:**

Compared with survivors (n = 289), non-survivors (n = 34) had higher levels of D1-UE-Cd. Stepwise multiple linear regression analysis indicated that D1-UE-Cd was positively associated with pulse rate and level of aspartate aminotransferase, but negatively associated with serum albumin level. Multivariate Cox analysis, with adjustment for other significant variables and measurements from mortality scoring systems, indicated that respiratory rate and D1-UE-Cd were independent and significant predictors of mortality. For each 1 μg/day increase of D1-UE-Cd, the hazard ratio for CCU mortality was 3.160 (95% confidence interval: 1.944–5.136, *p* < 0.001). The chi-square value of Hosmer-Lemeshow goodness-of-fit test for D1-UE-Cd was 10.869 (*p* = 0.213). The area under the receiver operating characteristic curve for D1-UE-Cd was 0.87 (95% confidence interval: 0.81–0.93).

**Conclusions:**

The D1-UE-Cd, an objective variable with no inter-observer variability, accurately predicted hospital mortality of CCU patients and outperformed other established scoring systems. Further studies are needed to determine the physiological mechanism of the effect of cadmium on mortality in CCU patients.

## Introduction

Cadmium is a well-known toxic metal [1], and occupational or environmental exposure is implicated in several clinical conditions, such as renal dysfunction, bone disease, and some cancers [2]. Notably, renal tubular damage may develop following exposure to lower levels of cadmium than previously anticipated [3]. Cadmium has a long half-life in humans (10–30 years) [2] and is excreted mainly in urine, normally less than 2 μg/day in persons without occupational exposure [4]. There is a positive correlation between urinary cadmium excretion and cadmium body burden [5], so the total daily urinary excretion of cadmium is a better indicator of cadmium accumulation than blood cadmium in humans [5,6].

In clinical practice, several scoring models are used to evaluate illness severity and predict prognosis, such as Sequential Organ Failure Assessment (SOFA), Acute Physiology and Chronic Health Evaluation-II (APACHE II), RIFLE (Risk, Injury, Failure, Loss, End-stage kidney Disease) criteria, Simplified Acute Physiology Score (SAPS), and the Multiple Organ Dysfunction Score [7–11]. Additionally, Lakkireddy et al. [12] developed a scoring model to quantify the outcomes of critically ill cardiac patients, the Modified Mid America Heart Institute Coronary Care Unit (CCU) scoring system. In spite of the widespread use and acceptance of these scoring models, there can be significant inter-observer variability in assessing individual patients [10,13]. Moreover, implementation of these scoring systems can be difficult and time-consuming. Recently, our research team showed that urinary cadmium excretion on day 1 (D1-UE-Cd) of admission to an intensive care unit (ICU) can predict illness severity and mortality of critically ill patients [6]. However, the association between urinary cadmium excretion and illness severity and mortality remains uncertain for critically ill patients admitted to CCUs. Furthermore, there is no single and reliable variable that accurately predicts hospital mortality in this population.

In this study, we examined the correlation between urinary cadmium excretion and outcome of critically ill cardiac patients admitted to a CCU and compared the results to existed scoring systems for predicting outcome.

## Methods

This clinical study was conducted in accordance with the Declaration of Helsinki and was approved by the Medical Ethics Committee of Chang Gung Memorial Hospital, a tertiary referral medical center in Taiwan. Written informed consent was obtained from every participant, and the study was approved by the Institutional Review Board of Chang Gung Memorial Hospital. All individual information was securely protected (by delinking identifying information from main data set) and available to investigators only. Furthermore, all the data were analyzed anonymously. This 6-month study examined the relationship between D1-UE-Cd with illness severity and mortality of critically ill patients admitted to a CCU.

### Patients and data collection

All study participants were from the CCU of the Chang Gung Memorial Hospital from Jan 1, 2005 to June 30, 2005 and were at least 18 years-old. Patients with any of the following criteria were excluded: total urine less than 500 mL on day 1 of CCU admission; duration of CCU stay less than 1 day; duration of hospital stay more than 150 days; presence of end-stage renal disease and undergoing maintenance dialysis; readmission to the CCU; and history of occupational, residential, or other exposure to cadmium or history of intoxication from other heavy metals. We collected the following data for analysis: demographic data, laboratory data, duration of CCU and hospital stays, pre-existing chronic diseases, initial diagnosis and clinical conditions upon CCU admission, and data for scoring predictive indices and patient outcomes. We recorded the total urine volume of patients during the first 24 h of CCU admission to calculate the amount of excretory cadmium in urine. All physicians who participated in this study were blinded to the results of D1-UE-Cd to reduce bias.

### Clinical scoring systems

We used 3 scoring models to evaluate the severity of illness based on data collected on day 1 of CCU admission: APACHE II, SOFA, and RIFLE. The scores of the APACHE II [14,15] and SOFA [8] were calculated as previously described. The RIFLE criteria, which evaluates the stage of acute kidney injury (AKI) as defined by the Acute Dialysis Quality Initiative (ADQI) Group [16], was based on the increase of serum creatinine and/or the decrease of glomerular filtration rate and urine output.

### Measurement of urinary cadmium

We collected urine samples in cadmium-free bottles and measured cadmium levels at 24 h after CCU admission. Urinary cadmium was measured as described by Jin et al. [17]. In particular, 500 μL of trace metal–grade distilled 0.8 M HNO_3_ and 100 μL of urine were added to 1.5-mL Eppendorf tubes and then immediately agitated. After overnight refrigeration, the vessels were warmed to room temperature, whirl-mixed for 5–10 s, and then centrifuged for 5 min at 11,500 rpm. The supernatant was transferred to graphite furnace sampler cups. The cadmium levels of the acid-digested samples were measured by electrothermal atomic absorption spectrometry (SpectrAA-220 Zeeman; Varian, Palo Alto, CA, USA) with Zeeman’s background correction and an L’vov platform. The detection limit for urinary cadmium concentration was 0.01 μg/L. Quality control was consistently confirmed by internal and external quality-control procedures. A certified commercially prepared product (Seronorm Trace Elements; Sero AS, Billingstads, Norway) was used to determine intra-batch accuracy and ensure inter-batch standardization. The coefficient of variation for the cadmium measurements was 5.0% or less. External quality control was maintained *via* participation in the National Quality Control Program conducted by the government of Taiwan.

### Statistical analysis

The Kolmogorov-Smirnov test was applied for the distribution of the continuous variables. Unless otherwise stated, continuous variables were expressed as mean ± standard deviation or median with interquartile range (IQR), and categorical variables were expressed as number with percentage. We used the chi-square test or Fisher’s exact test to compare categorical data of survivors and non-survivors, and Student’s *t-*test or Mann-Whitney *U* test to identify the significance of differences between groups. To identify factors associated with D1-UE-Cd, we used a simple linear regression analysis for all variables. All potential variables (*p* < 0.05) from this simple linear regression were entered into multiple linear regression models with backward stepwise procedures. Data were expressed as standardized regression coefficients (β) and *p* values in the linear regression analysis.

We used the Cox proportional-hazard model to assess the effect of baseline variables on mortality, hazard ratios (HRs), and 95% confidence intervals (CIs) of mortality. A univariate Cox model was used to identify the association of all variables with mortality; variables with *p-*values less than 0.05 were entered into a multivariate Cox model with a forward stepwise procedure.

We used the Hosmer-Lemeshow (HL) test to assess goodness-of-fit in the models [18]. We applied receiver operating characteristic curve (ROC) to evaluate discrimination, the ability of the model to distinguish between death and survival, by assessing the area under ROC (AUROC) [19,20]. All statistical tests were two tailed, and a *p*-value less than 0.05 was considered statistically significant. Data were analyzed using StatView 2.0 for Windows (SAS Institute, Cary, NC) and SPSS version 18.0 for Windows XP (SPSS, Chicago, IL).

## Results

### Patient characteristics

A total of 323 critically ill cardiac patients (209 males and 114 females) in the CCU met our enrollment criteria and had complete medical records (Table 1). The mean patient age was 65.3 ± 15.0 years; mean body mass index (BMI) was 24.29 ± 4.57 kg/m^2^. 151 patients (46.7%) were smokers. The median duration in the CCU was 4.0 days (IQR: 3.0–8.0), and the median duration of hospitalization was 11.0 days (IQR: 7.0–27.0). The median APACHE score was 10.0 (IQR: 5.75–17.0). The median SOFA score was 3.0 (IQR: 1.0–6.0), and the median D1-UE-Cd was 0.34 μg/day (IQR: 0.15–0.80). The 3 main causes of CCU admission were acute myocardial infarction (46.4%, n = 150), class III/IV congestive heart failure (CHF) (24.8%, n = 80), and arrhythmia (9.6%, n = 31). The overall mortality rate was 10.5% (n = 34).

**Table 1:** Baseline characteristics of study patients upon admission to the cardiac care unit (n = 323).

Variable	Survivors (n = 289)	Non-survivors (n = 34)	*P*
**Demographic data**			
Age (years)	64.9 ± 14.6	68.9 ± 16.5	0.140
Sex (male)	195 (67.5)	14 (41.2)	0.004
Body mass index (kg/m^2^)	24.48 ± 4.6	22.64 ± 3.95	0.027
Smoking (Yes)	143 (49.5)	8 (23.5)	0.006
CCU stay (days)	4.0 (3.0–7.0)	11.0 (6.3–28.5)	<0.001
Hospital stay (days)	11.0 (7.0–24.0)	19.5 (10.8–32.0)	0.028
**Co-morbidities**			
Previously diagnosed cardiovascular disease	128 (44.3)	21 (61.8)	0.068
Previously diagnosed chronic kidney disease	48 (16.6)	13 (38.2)	0.005
Previously diagnosed chronic pulmonary disease	31 (10.7)	5 (14.7)	0.561
Hyperlipidemia	126 (43.6)	11 (32.4)	0.271
Diabetes mellitus	99 (34.3)	14 (11.8)	0.450
Hypertension	166 (57.4)	16 (47.1)	0.276
**Diagnosis and clinical condition on admission**			
Acute myocardial infarction	138 (47.8)	12 (35.3)	0.152
CHF class III and IV	67 (23.2)	13 (38.2)	0.037
Arrhythmia	28 (9.7)	3 (8.8)	1.000
Unstable angina	25 (8.7)	0 (0.0)	0.091
Aortic dissection	10 (3.5)	0 (0.0)	0.608
Post-CPCR	4 (1.4)	2 (5.9)	0.130
Hypertension crisis	3 (1.0)	1 (2.9)	0.369
Infective endocarditis	3 (1.0)	1 (2.9)	0.369
Other	11 (3.8)	2 (5.9)	0.640
Circulatory shock	20 (6.9)	15 (44.1)	0.001
Acute respiratory failure	72 (24.9)	15 (44.1)	0.024
AKI	30 (10.4)	16 (47.1)	<0.001
**Predictive indices on admission**			
APACHE II score	9.0 (5.0–16.0)	20.5 (15.3–25.0)	<0.001
SOFA score	2.0 (1.0–5.3)	7.0 (4.3–9.8)	<0.001
RIFLE criteria (class F)	9 (3.1)	4 (11.8)	<0.001
D1-UE-Cd (μg/day)	0.30 (0.14–0.63)	1.53 (0.80–3.07)	<0.001

Data presented as mean ± standard deviation, number (percentage), and median (interquartile range).

Cardiovascular diseases included stroke, ischemic heart disease, valve diseases and peripheral vascular diseases. Chronic kidney disease defined as persistent abnormal renal function (serum creatinine > 1.4 mg/dL) for 6 months at least. Chronic pulmonary diseases included asthma, chronic bronchitis, chronic obstructive pulmonary disease and lung fibrosis. Hyperlipidemia defined as diagnosed by a physician and required regular treatments with antilipidemic agents. Diabetes mellitus was diagnosed by a physician and required regular treatments with antihyperglycemic drugs. Hypertension defined as blood pressure > 140/90 mm Hg at least twice measurements and required regular treatments with antihypertensive drugs. Shock defined as mean arterial pressure < 60 mm Hg. Acute respiratory failure defined as acute onset of respiratory failure required ventilator support. Acute kidney injury defined as serum creatinine >2.0 mg/dL and/or daily urine amount <500 mL.

**Abbreviations here and below:** AKI, acute kidney injury; APACHE, Acute Physiology and Chronic Health Evaluation; CCU, coronary care unit; CHF, congestive heart failure; CPCR, cardiopulmonary cerebral resuscitation; D1-UE-Cd, day 1 urinary excretion of cadmium; RIFLE, Risk, Injury, Failure, Loss, End-stage kidney Disease; SOFA, Sequential Organ Failure Assessment.

Table 1 compares the baseline characteristics of survivors (n = 289) and non-survivors (n = 34). Non-survivors were more likely to have previously diagnosed chronic kidney disease, AKI, class III/IV CHF, circulatory shock, and acute respiratory failure. Survivors were more likely to be male, smokers, and have a higher BMI. Furthermore, non-survivors had longer CCU and hospital stays, higher APACHE II score, SOFA score, and RIFLE score, and higher levels of D1-UE-Cd.

Analysis of vital signs and biochemical data indicated that non-survivors had higher arterial pressure, pulse and respiratory rates, and levels of aspartate aminotransferase (AST), blood urea nitrogen (BUN), and creatinine. Survivors had higher scores on the Glasgow coma scale and higher levels of serum albumin (Table 2).

**Table 2:** Baseline vital signs and biochemical data of study patients upon admission to the cardiac care unit (n = 323).

Variable	Survivors (n = 289)	Non-survivors (n = 34)	*P*
**Vital signs**			
Glasgow Coma scale	13.6 ± 3.3	11.6 ± 4.2	0.011
Mean arterial pressure (mm Hg)	76.8 ± 15.9	86.7 ± 18.0	0.003
Body temperature (°C)	36.9 ± 0.8	37.1 ± 1.2	0.186
Pulse rate (beats/min)	83.8 ± 22.1	100.2 ± 25.2	<0.001
Respiratory rate (breaths/min)	19.8 ± 5.0	22.7 ± 6.4	0.002
**Biochemical data**			
PaO_2_/FiO_2_ (mm Hg)	274.1 ± 146.1	271.9 ± 175.4	0.938
Albumin (g/dL)	3.43 ± 0.53	3.01 ± 0.55	<0.001
AST (IU/L)	38.0 (25.0–82.0)	51.0 (30.0–427.0)	0.017
ALT (IU/L)	31.0 (19.0–58.3)	42.0 (14.0–239.0)	0.276
Total bilirubin (mg/dL)	1.12 ± 1.60	1.00 ± 0.86	0.416
BUN (mg/dL)	31.0 ± 24.8	45.1 ± 28.9	0.010
Creatinine (mg/dL)	1.55 ± 1.23	2.12 ± 1.36	0.015
Sodium (mmol/L)	138.6 ± 7.0	139.0 ± 6.4	0.722
Potassium (mmol/L)	3.96 ± 0.63	3.97 ± 0.89	0.964
Hemoglobin (g/dL)	13.3 ± 6.5	11.1 ± 3.2	0.055
White blood cells (10^3^/μL)	10.8 ± 4.2	12.8 ± 5.8	0.054
Platelets (10^3^/μL)	209.3 ± 84.2	204.3 ± 81.3	0.740
Cardiac ejection fraction (%)	52.9 ± 18.3	46.8 ± 17.8	0.064

Data presented as mean ± standard deviation and median (interquartile range).

**Abbreviations here and below:** AST, aspartate aminotransferase; ALT, alanine aminotransferase; BUN, blood urea nitrogen.

### Determinants of urinary cadmium excretion on day 1 of CCU admission

Simple linear regression analysis (Table 3) indicated that D1-UE-Cd was positively associated with circulatory shock, pulse and respiratory rates, AST, white blood cell count, APACHE II and SOFA scores; however, D1-UE-Cd was negatively associated with sex, BMI, hyperlipidemia, hypertension, Glasgow coma scale score, mean arterial pressure, serum albumin, hemoglobin, and cardiac ejection fraction. After adjusting for potential confounding, multiple linear regression analysis with backward stepwise procedures indicated that pulse rate (β = 0.202, *p* = 0.002) and AST level (β = 0.140, *p* = 0.027) were positively associated with D1-UE-Cd, and that albumin level (β = -0.258, *p* < 0.001) was negatively associated with D1-UE-Cd.

**Table 3:** Determinants of urinary cadmium excretion on day 1 of CCU admission in all study patients

Variable	Simple Linear Regression β	*P*	Backward Stepwise Multiple Linear Regression β	*P*
Sex	-0.124	0.044		
Body mass index (kg/m^2^)	-0.175	0.004		
Hyperlipidemia	-0.171	0.005		
Hypertension	-0.146	0.017		
Shock	0.233	<0.001		
Glasgow Coma scale	-0.228	<0.001		
Mean arterial pressure	-0.174	0.004		
Pulse rate (beats/min)	0.256	<0.001	0.202	0.002
Respiratory rate (breaths/min)	0.143	0.019		
Albumin (g/dL)	-0.308	<0.001	-0.258	<0.001
AST (IU/L)	0.171	0.012	0.140	0.027
Hemoglobin (g/dL)	-0.172	0.005		
White blood cells (10^3^/μL)	0.236	<0.001		
Cardiac ejection fraction (%)	-0.122	<0.001		
APACHE II score	0.354	<0.001		
SOFA score	0.291	<0.001		

Data were expressed as standardized regression coefficients (β) and *p* values.

### Cox regression analysis for mortality of the CCU patients

Univariate Cox regression analysis identified 20 potential predictors of mortality (Table 4), including sex, smoking, BMI, AKI, shock on admission, score of the Glasgow coma scale, mean arterial pressure, pulse and respiratory rates, serum albumin levels, AST, alanine aminotransferase, BUN, creatinine, hemoglobin, white blood cell count, APACHE II score, SOFA score, RIFLE score, and D1-UE-Cd.

**Table 4:** Univariate Cox analysis of risk factors for hospital death in all study patients according to baseline variables at the first day in the cardiac care unit. (*P >*0.05 to remove)

Variable	HR (95% CI)	*P*
Sex (female *vs*. male)	2.804 (1.385–5.679)	0.004
Smoking (Yes *vs*. no)	3.299 (1.427–7.628)	0.005
Body mass index (Increase of 1 kg/m^2^)	0.883 (0.805–0.969)	0.008
AKI on admission (Yes *vs*. no)	3.228 (1.173–8.885)	0.023
Previously diagnosed chronic kidney disease (Yes *vs*. no)	2.847 (1.389–5.836)	0.004
CHF class III and IV (Yes *vs*. no)	3.307 (1.704–6.419)	<0.001
Mean arterial pressure (Increase of 1 mm Hg)	0.972 (0.952–0.993)	0.009
Pulse rate (Increase of 1 beat/min)	1.026 (1.013–1.039)	<0.001
Respiratory rate (Increase of 1 breath/min)	1.080 (1.029–1.134)	0.002
Albumin (Increase of 1 g/dL)	0.237 (0.112–0.458)	<0.001
AST (Increase of 1 U/L)	1.001(1.000–1.002)	0.001
ALT (Increase of 1 U/L)	1.001(1.000–1.002)	0.035
BUN (Increase of 1 mg/dl)	1.014 (1.005–1.023)	0.003
Creatinine (Increase of 1 mg/dL)	1.289 (1.082–1.535)	0.004
Hemoglobin (Increase of 1 g/dL)	0.786 (0.698–0.886)	<0.001
White blood cells (Increase of 10^3^/μL)	1.068 (1.002–1.139)	0.044
APACHE II score (Increase of 1)	1.107 (1.070–1.146)	<0.001
SOFA score (Increase of 1)	1.247 (1.152–1.349)	<0.001
RIFLE score (Increase of 1)	1.609 (1.222–2.119)	0.001
D1-UE-Cd (Increase of 1 μg/day)	1.751 (1.514–2.025)	<0.001

**Abbreviations here and below:** HR: Hazard Ratio; CI: confidence interval.

We determined the independent effect of each predictor by entering these data into a multivariate Cox regression analysis with a forward stepwise method (Table 5). The results show that respiratory rate and D1-UE-Cd were independent predictors of mortality. The HR of CCU mortality for each increment of 1 breath/min was 1.139 (95% CI: 1.056–1.228, *p* = 0.001) and the HR of CCU mortality for each increment of 1μg/day of urinary cadmium was 3.160 (95% CI: 1.944–5.136, *p* < 0.001).

**Table 5:** Forward multivariate Cox analysis of risk factors for hospital death in all study patients, according to baseline variables at the first day in the cardiac care unit. (*P* > 0.1 to remove)

Variable	HR (95% CI)	*P*
Respiratory rate (Increase of 1 breath/min)	1.139 (1.056–1.228)	0.001
D1-UE-Cd (Increase of 1 μg/day)	3.160 (1.944–5.136)	<0.001

### Analysis of goodness-of-fit and ROC curves for scoring systems and urinary cadmium levels

We measured goodness-of-fit of these models by use of the HL chi-square test and obtained the following results: (1) APACHE II (HL chi-square = 6.584, 8 degrees of freedom [*df*], *p* = 0.582) (2) SOFA (HL chi-square = 5.561, 7 *df*, *p* = 0.592) (3) RIFLE (HL chi-square = 0.001, 1 *df*, *p* = 1.000) (4) D1-UE-Cd (HL chi-square = 10.869, 8 *df*, *p* = 0.213).

**Fig 1:**
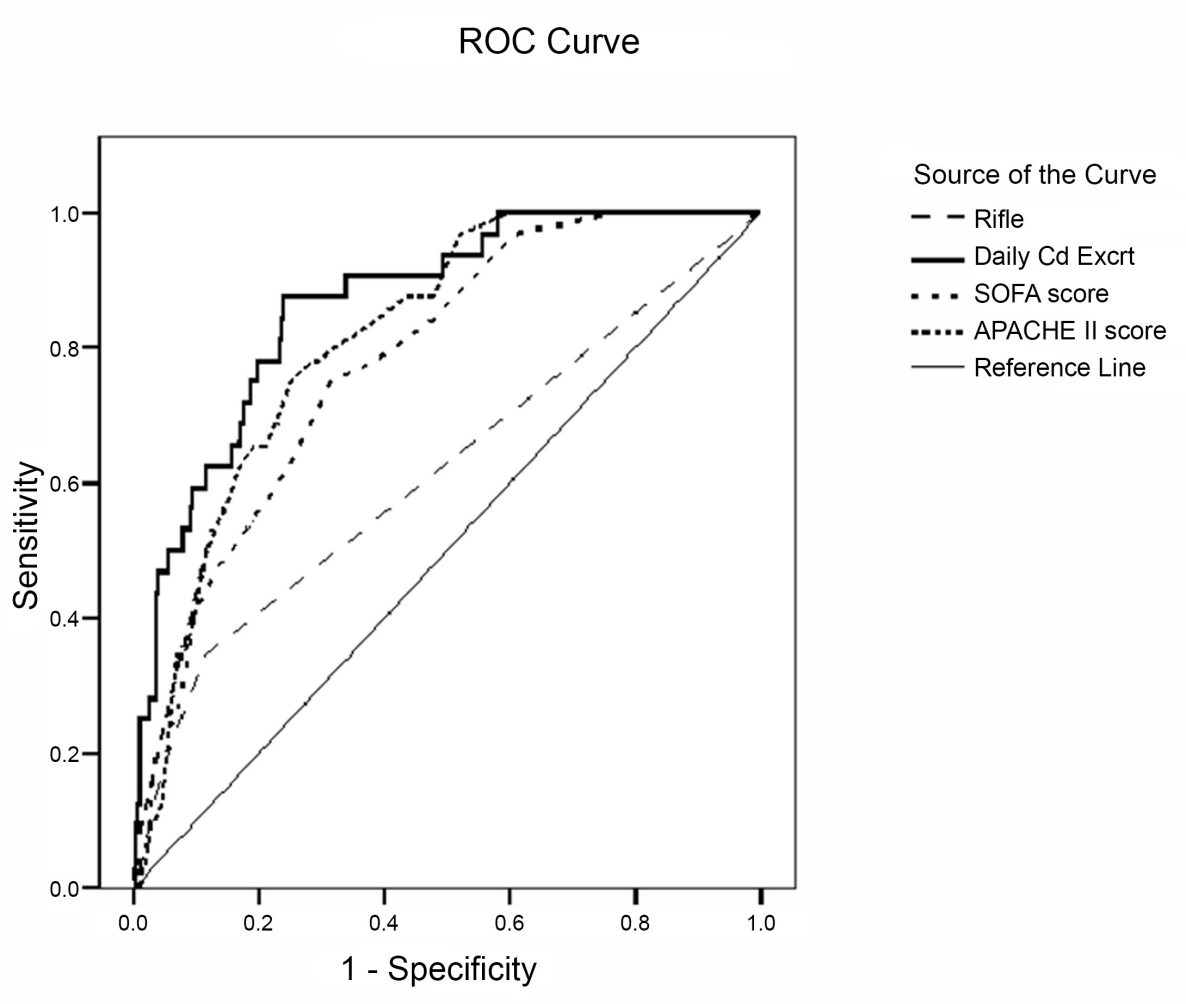
Receiver operating characteristic (ROC) curves based on coronary care unit (CCU) day 1 urinary cadmium excretion (area under ROC [AUROC] = 0.87), Acute Physiology and Chronic Health Evaluation (APACHE) II score (AUROC = 0.82), Sequential Organ Failure Assessment (SOFA) score (AUROC = 0.78) and Risk, Injury, Failure, Loss, End-stage kidney Disease (RIFLE) criteria (AUROC = 0.62).

We also used these data for ROC analysis (Fig 1). Computation of the area under the ROC curves (AUROCs) indicated that D1-UE-Cd (AUROC = 0.87 ± 0.03, 95% CI: 0.81–0.93, *p* < 0.001) had better discriminatory power than the APACHE II score (AUROC = 0.82 ± 0.03, 95% CI: 0.75–0.88, *p* < 0.001), SOFA score (AUROC = 0.78 ± 0.04, 95% CI: 0.71–0.86, *p* < 0.001), or RIFLE criteria (AUROC = 0.62 ± 0.06, 95% CI: 0.50–0.73, *p* < 0.001).

## Discussion

The results of this 6 month study of mortality in patients at a CCU in Taiwan demonstrated that non-survivors had longer CCU and hospital stays, higher APACHE II, SOFA, and RIFLE scores, and greater D1-UE-Cd. Moreover, more non-survivors had previously diagnosed chronic kidney disease, class III and IV CHF, circulatory shock, acute respiratory failure, and AKI upon CCU admission; non-survivors also had higher mean arterial pressure, pulse and respiratory rates, and levels of AST, BUN, and serum creatinine. Non-survivors were less likely to be male, less likely to smoke, and had lower BMI, Glasgow coma scale score, and serum albumin level. Notably, D1-UE-Cd was significantly associated with mortality after adjusting for other variables by multivariate Cox regression analysis, including APACHE II, SOFA, and RIFLE scores. The HR of mortality associated with each 1 μg/day increase of urinary cadmium was 3.160. Hence, D1-UE-Cd appears to be a good predictor of hospital mortality in critically ill cardiac patients; the other findings of the present study are similar to the results of previous studies of CCU patients [21,22]. This is the first study to demonstrate that urinary cadmium excretion is significantly associated with mortality in CCU patients.

Many scoring systems [7–11] have been developed to describe the severity of illness in ICU patients and to predict patient outcome following medical care. The APACHE II [10] and SAPS II scores [7] are based a patient’s clinical status during the first 24 h of ICU admission, and the SOFA score [8] quantifies illness severity based on the function or failure rate of different organ system. Although these scoring systems are widely used, they can be complicated and time-consuming in clinical practice. Moreover, use of these scoring systems requires training and adherence to strict guidelines [10], because inter-observer variability can occur when different physicians evaluate the same patient [10,23]. Hence, a simple predictor is needed for critical care patients because more aggressive treatments may be required if there is increased risk of mortality. Compared with these scoring systems, the D1-UE-Cd (recorded upon CCU admission), is a simple and useful predictor of mortality in patients admitted to the CCU. However, more studies are needed to the clinical suitability of this test before it can be widely accepted as an independent predictor of mortality in CCU patients.

Previous studies of general populations have examined the association between urinary cadmium and mortality. For example, Menke et al. [24] demonstrated that environmental cadmium exposure increased the risk of death from all-causes, all cancers, and cardiovascular disease among U.S. men who participated in the Third National Health and Nutrition Examination Survey in 1988–1994. Another study by Cheung et al. [25] showed that urinary cadmium was a predictor of mortality from all-causes, all cancers, and prostate cancer in men, based on data of National Health and Nutrition Examination Survey (NHANES III). Moreover, a 22-year follow-up study by Li et al. [26] of a cadmium-polluted area in Japan suggested a dose-response relationship between cadmium body burden and mortality from cardiovascular diseases, cerebrovascular diseases, and nephritis in the 3119 inhabitants. However, the present study is the first to report a significant association between body cadmium level and mortality in critically ill cardiac patients who have no history of cadmium exposure.

Although the cause of the increased cadmium level in the biological fluids of CCU patients remains uncertain, several *in vitro* and *in vivo* studies provide some insight into the cardiac effects of cadmium exposure. In rats, Ferramola et al. [27,28] demonstrated that cadmium may induce myocardial injury by increasing oxidative stress. Ozturk et al. [29] demonstrated that cadmium intoxication can cause deformation of cardiac muscle cells due to an increase of free radicals and lipid peroxidation. In humans, Ponteva et al. [30] showed that the mean blood cadmium level of 47 patients with myocardial infarction was significantly higher than that of 37 control subjects. Smetana et al. [31] reported that 54 patients with dilated cardiomyopathy had higher blood and urinary levels of cadmium than 17 healthy controls. Moreover, Tellez-Plaza et al. [32] performed a prospective cohort study of 3348 adults and showed that elevated urinary cadmium was associated with increased cardiovascular mortality and cardiovascular disease. Taken together with our findings, this suggests that cadmium may play a role in the pathogenesis of cardiovascular diseases. However, further studies are needed to elucidate the mechanism by which cadmium increases the mortality of CCU patients.

In ROC analysis, a parameter with an AUROC of 0.80 or more is considered a good predictor [7,33], and a parameter with an AUROC of 0.70 or less is considered a poor predictor [20,34]. In the present study, our ROC analysis indicated that D1-UE-Cd had the greatest AUROC (0.87 ± 0.03, 95% CI: 0.81–0.93), and was the only parameter whose 95% CI is entirely above 0.80. Moreover, comparison of ROC curves demonstrated that D1-UE-Cd outperformed the SOFA, APACHE II, and RIFLE scoring systems (Fig 1). Furthermore, we performed model calibration by the HL goodness-of-fit test, which determines how well the predicted outcomes match the observed outcomes throughout a range of risks [18]. An HL chi-square less than 15 and a *p*-value of 0.2–0.8 are considered acceptable [13,35]; thus, our analysis indicated that D1-UE-Cd had good calibration (HL chi-square = 10.869, *p* = 0.213). All of these findings indicate that D1-UE-Cd has high discrimination and calibration in the prediction of mortality for CCU patients, and that it appears to be superior to the SOFA, APACHE II, and RIFLE scoring systems. Hence, D1-UE-Cd is a simple and reliable predictor of mortality in this population. Further studies are needed to examine whether D1-UE-Cd can also be used as an indicator of response to treatment among CCU patients.

Previous studies reported that an elevated level of AST [36] and a lower level of serum albumin [37,38] occur in patients with severe cardiac complications, such as acute myocardial infarction or heart failure. The current study, after adjusting for potential variables by multiple linear regression analysis with backward stepwise procedures, demonstrated that D1-UE-Cd was positively associated with AST and negatively associated with serum albumin. As a nonspecific biomarker of myocardial injury, AST may be elevated in critically ill cardiac patients [39]. Cadmium usually accumulates in the liver and kidney [1], and patients with critical cardiac complications (*e*.*g*. acute myocardial infarction, class III or IV congestive heart failure, arrhythmia, unstable angina, and aortic dissection) may suffer from severe liver and kidney damage, so damage of these organs may explain the higher level of urinary cadmium in more severely ill patients [6]. Moreover, liver damage may decrease the synthesis of serum albumin in humans. An *in vitro* study [40] also reported that cadmium (a potent nephrotoxin), can lead to albuminuria by impairing reabsorption and secretion in the renal proximal tubule. However, further studies are needed to verify the mechanisms underlying the relationships of D1-UE-Cd, serum AST, and serum albumin in CCU patients.

There are several limitations of this study. Although the results indicated that D1-UE-Cd can predict hospital mortality in critically ill cardiac patients, the mechanism underlying this relationship is still unclear. Hence, more studies are needed to explore the physiological dynamics of cadmium and what D1-UE-Cd means in this population, and some studies are underway in our research center. Furthermore, the enrolled patients were not an incident cohort of patients with critical cardiac events, so there may have been survivorship bias. However, the association between D1-UE-Cd and mortality remained after adjustment for potential confounding in our multivariate Cox analysis. Additionally, our sample size was somewhat small and the study was performed at a single institution, so the results may not be applicable to CCU patients of other hospitals. Hence, further large-scale multi-center studies are required to confirm our observations.

## Conclusions

In summary, this study is the first to demonstrate that D1-UE-Cd in patients admitted to the CCU is independently associated with mortality. This index has good calibration and discrimination, and that it outperformed the APACHE II, SOFA, and RIFLE scoring systems by ROC analysis. Moreover, compared with other scoring systems, measurement of D1-UE-Cd is a single objective variable, and there is no inter-observer variability among physicians. Because of the small number of patients in the present study, the predictive value of cadmium in CCU patients needs further validation. Moreover, additional studies are needed to establish the mechanism by which cadmium exposure increases cardiovascular mortality.
